# 3D‐Laminated Graphene with Combined Laser Irradiation and Resin Infiltration toward Designable Macrostructure and Multifunction

**DOI:** 10.1002/advs.202200362

**Published:** 2022-03-24

**Authors:** Yan Gao, Yujiang Zhai, Guantao Wang, Fu Liu, Haibin Duan, Xilun Ding, Sida Luo

**Affiliations:** ^1^ School of Mechanical Engineering & Automation Beihang University No. 37 Xueyuan Road Beijing 100191 China; ^2^ School of Automation Science and Electrical Engineering Beihang University No. 37 Xueyuan Road Beijing 100191 China

**Keywords:** 3D graphene, graphene papers, laser‐induced graphene, multifunctional sensors, smart composites

## Abstract

Macroscopic 3D graphene has become a significant topic for satisfying the continuously upgraded smart structures and devices. Compared with liquid assembling and catalytic templating methods, laser‐induced graphene (LIG) is showing facile and scalable advantages but still faces limited sizes and geometries by using template induction or on‐site lay‐up strategies. In this work, a new LIG protocol is developed for facile stacking and shaping 3D LIG macrostructures by laminating layers of LIG papers (LIGPs) with combined resin infiltration and hot pressing. Specifically, the constructed 3D LIGP composites (LIGP‐C) are compatible with large area, high thickness, and customizable flat or curved shapes. Additionally, systematic research is explored for investigating critical processing parameters on tuning its multifunctional properties. As the laminated layers are stacked from 1 to 10, it is discovered that piezoresistivity (i.e., gauge factor) of LIGP‐C dramatically reflects an ≈3900% improvement from 0.39 to 15.7 while mechanical and electrical properties maintain simultaneously at the highest levels, attributed to the formation of densely packed fusion layers. Along with excellent durability for resisting multiple harsh environments, a sensor‐array system with 5 × 5 LIGP‐C elements is finally demonstrated on fiber‐reinforced polymeric composites for accurate strain mapping.

## Graphical abstract

Macroscopic 3D graphene is innovatively created through combined laser irradiation and resin infiltration processes with large area (≈400 cm^2^), high thickness (≈3.5 mm), and customizable flat/curved geometries. With hot‐press‐induced fusion layers, the 3D graphene is unique for simultaneously acquiring high strength (24.7 MPa), electrical conductivity (143 S m^−1^), and gauge sensitivity (15.7).

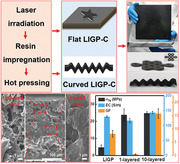

## 1 Introduction

Graphene, a honeycomb‐lattice‐structured molecule owning superb specific surface area (SSA, ≈3500 m^2^ g^−1^),^[^ ^1^ ^]^ tensile strength (≈130 GPa),^[^ ^2^ ^]^ thermal conductivity (≈5000 W m^−1^ K^−1^),^[^ ^3^ ^]^ bulk conductivity (≈303 S m^−1^), and intrinsic carrier mobility (10^5^–10^7^ cm^2^ V^−1^ s^−1^),^[^ ^4^, ^5^ ^]^ has drawn tremendous attention from academia to industries.^[^ ^4^, ^6^ ^]^ When applied practically, the nanosized graphene particles always need to be assembled together as certain format of macrostructures to support key structures and functions for various smart devices and systems, such as working electrodes of micro‐supercapacitors and sensing elements of flexible electronics.^[^ ^7^, ^8^ ^]^ In comparison with 1D (e.g., graphene‐based filaments, yarns, and composite fibers)^[^ ^9^, ^10^, ^11^ ^]^ and 2D (e.g., graphene‐based thin films, papers, and fabrics)^[^ ^12^, ^13^ ^]^ geometries with small, thin, and/or binder/substrate‐supported natures, the graphene‐based 3D aerogels,^[^ ^14^ ^]^ hydrogels,^[^ ^15^ ^]^ foams,^[^ ^16^ ^]^ millispheres,^[^ ^17^ ^]^ and laminated structures/composites^[^ ^18^, ^19^ ^]^ were also intriguingly focused as another important macroscopic ensembles, exhibiting multiple unique characteristics including free‐standing and/or binder‐free structures, widely scaled and highly designable configurations, and organized networking architectures with interconnected nanoparticles/ domains.^[^ ^20^ ^]^ Hence, the building of macroscopic 3D graphene has become a significant topic for satisfying the continuously upgraded structures and functions in various novel devices, such as artificial skins and muscles, wearable electronics, biomimetic surfaces, thin‐film batteries, and soft robots.^[^ ^21^, ^22^, ^23^, ^24^ ^]^

To fabricate 3D graphene macrostructures, current processing strategies have basically been categorized into liquid assembling and catalytic templating methods. The former process generally relies on the self‐assembly of graphene oxide (GO) transformed from liquid dispersion to 3D self‐supported solid networks. For examples, Xu et al.^[^ ^25^ ^]^ and Wang and Ellsworth,^[^ ^26^ ^]^ respectively, applied hydrothermal and freeze‐drying plus annealing methods to cast and reduce GO dispersion to form cylindrical‐shaped graphene hydrogels or aerogels with centimeter‐scaled volume. As another type of liquid assembling, 3D printed techniques including inkjet layer‐by‐layer printing,^[^ ^27^, ^28^ ^]^ extrusion printing,^[^ ^29^, ^30^ ^]^ and stereolithography^[^ ^31^ ^]^ are able to process graphene macrostructures or devices with more complex and even customized shapes. Nevertheless, the above‐mentioned liquid assembling methods overall face issues of complicated and prolonged steps from dispersion preparation (including sonication, ultracentrifugation, chemical stabilization, etc.), structure molding (including ice‐templating, wet‐spinning, solution‐casting, etc.), to post‐treatments (including chemical reduction, high‐temperature annealing, interfacial transferring, etc.),^[^ ^32^, ^33^, ^34^, ^35^ ^]^ in which the multiple correlated parameters could further enhance the difficulty to control the uniformity of particle dimension, microstructure, chemical composition, and physical/chemical characteristics. By avoiding the process to disperse and reduce GO, the catalytic templating method relies on catalytic decomposition of hydrocarbons on 3D templates to synthesize graphene monoliths.^[^ ^36^ ^]^ For examples, Cheng et al.^[^ ^36^ ^]^ and Chen et al.,^[^ ^37^ ^]^ respectively, used chemical vapor deposition (CVD) and plasma‐enhanced CVD to decompose gaseous hydrocarbon precursor to grow foam‐like graphene structures on 3D nickel‐foam templates with the size of 20 × 20 × 2 mm^3^. Similar to liquid assembling, the bottom‐up synthesis also involves multistepped chemical and physical processes (e.g., template formation and etching, precursor pyrolysis and deposition) with ultrahigh temperature (≈1000 °C), rigorous atmosphere control (Ar or H_2_ as carrier gas), and confined space (e.g., quartz tube with ≈25 mm diameter).^[^ ^36^, ^37^ ^]^ With the above common issues to inevitably induce the graphene macrostructure with limited sizes, shapes, productivity, and homogeneity, a new protocol is highly anticipated with higher‐leveled processing efficiency, size scalability, and characteristic tunability.

Until recently, laser‐induced graphene (LIG) has become a low‐cost, facile, and scalable process for converting and assembling graphene structures or devices from certain polymeric, natural biomass, or nonpolymeric precursors through laser‐irradiation‐induced photothermal treatment.^[^ ^38^, ^39^, ^40^, ^41^ ^]^ By utilizing computer‐aided design and manufacture (CAD/CAM) based modes with customizable working routes, pioneering works have explored the capability of LIG for assembling various macroscopic graphene structures, e.g., LIG enabled fibers,^[^ ^42^ ^]^ thin films,^[^ ^43^ ^]^ papers,^[^ ^44^, ^45^, ^46^ ^]^ and foams.^[^ ^47^, ^48^ ^]^ Among which, the LIG‐based 1D or 2D structures always rely on a fast formation by directly processing the precursor with identical fiber‐ or film‐like shapes without the necessity of pre‐ or post‐treatments. The 3D‐based structures, nevertheless, are inevitably required the assistance of additional processes/treatments to assure the structural integrity when forming the macrostructure layer‐by‐layer. For example, Sha et al.^[^ ^47^ ^]^ applied sucrose and nickel mixed nanoparticles as both the precursor of LIG and 3D template for constructing 1 × 1 × 0.8 cm^3^ sized 3D foams. Although effectively avoiding the issue of lengthy and high‐temperature processing, the etching of Ni template is indispensable in which it causes ≈48.8% volume shrinkage. Alternatively, Luong et al.^[^ ^48^ ^]^ developed a laminated object process to manufacture centimeter‐sized LIG monoliths with repeated layer‐bonding and binder‐decomposing processes. Although compatible with automated setup, the hybrid fusing, milling, and annealing processes relying on multiple laser sources require the on‐site lay‐up of different layers one by one, still introducing the bulk foam with limited size (≈1 cm^3^) and elastic modulus ≈300 kPa.

Based on current strategies, two critical aspects should be continuously focused for further escalating the promising LIG for assembly of 3D macrostructures. The first one is to improve the efficiency and scalability for laying and shaping; the second one is to understand the process‐related performance for suiting multiscenario applications. Following this line of thought, in this work we developed a new LIG protocol by laminating layers of laser‐induced graphene papers (LIGPs) with combined resin infiltration and hot pressing. Compared with previous methods relying on template induction and on‐site lay‐up, the preparation of freestanding LIGP layers with high‐efficient infiltration of AG80 resin is unique for facile stacking and shaping 3D LIG macrostructures with scalable sizes and variable shapes. Accordingly, the constructed 3D LIGP/epoxy laminated composites (LIGP‐C) are compatible with large area (≈400 cm^2^), high thickness (≈3.5 mm), and customizable flat or curved configurations. Based on the unique process, systematic research was then explored for investigating multiple processing parameters on tuning three key properties of the 3D LIGP‐C, including tensile strength, electrical conductivity, and piezoresistivity. For single‐layered structures, under optimal lasing conditions, resin content (from 5 to 35 wt%) was found to be determinant for monotonically improving the tensile strength from 5.8 to 23.7 MPa while keeping the electrical conductivity at high levels (≈120 S m^−1^). As the laminated layer number was accumulated from 1 to 10, an interesting phenomenon was further discovered that the piezoresistive property (i.e., gauge factor) of 3D LIGP‐C dramatically reflects an ≈3900% improvement from 0.39 to 15.7, attributed to the formation of densely packed fusion layers under hot‐pressing process. With the established process–structure–property relationship, it is strongly advisable that the 3D LIGP‐C with multilayered configuration is easy to simultaneously acquire high‐leveled mechanical, electrical, and sensing performance. Benefit from the excellent properties along with the durability for resisting multiple harsh environments, a sensor‐array system with 5 × 5 LIGP‐C elements was successfully designed and manufactured on fiber‐reinforced polymer (FRP) composites for mapping various strain distributions of the host structure.

## 2 Result and Discussion

### 2.1 Combined Laser‐Irradiation, Resin‐Impregnation, and Hot‐Pressing Processes of 3D LIGP‐C with Diversified Macrostructures

**Figure 1:**
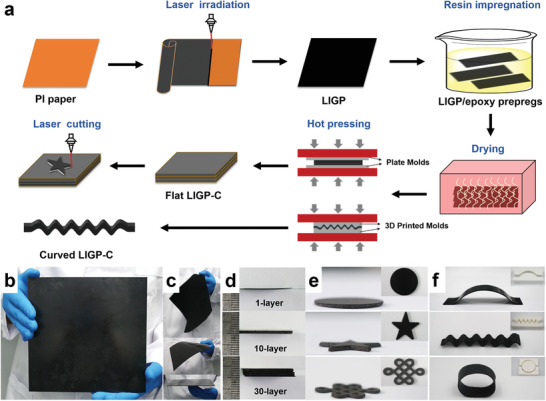
a) Schematic illustration of 3D LIGP‐C fabrication. Photographs of b) 20‐layered LIGP‐C, c) LIGP and single‐layered LIGP‐C with 20 × 20 cm^2^. Demonstrations of 3D LIGP‐C with d) different thicknesses and e ‐ f) various shapes achieved by laser cutting or 3D‐printed molds.

As schematically depicted in **Figure** **1**a, the developed manufacturing of 3D LIGP/epoxy laminated composites (LIGP‐C) includes three key processing steps, i.e., laser irradiation, resin impregnation, and hot pressing. The laser irradiation process (detailed in the Experimental Section) guarantees the swift and scalable formation of freestanding LIGPs, serving as the sheet‐typed raw materials for lamination. By applying highest laser scribing speed (≈30 cm^2^ min^−1^), a 1400 cm^2^ LIGP (the maximum working size of laser system) can be produced in 47 min; by upgrading the system for continuous process, a 5 m long LIGP has been achieved, as shown in Figure S1a (Supporting Information). With large size and high efficiency, it is also feasible for trimming the lamination layer with any desired dimensions using higher leveled laser (2.5 W). The resin impregnation by resolving the epoxy in the acetone solution with varied weight percentage (5–35 wt%) is then a controllable process to adjust the amount of infiltrated resin (0.08–0.66 g cm^−3^) merged with the laminated structure (Figure S2, Supporting Information). By providing a high‐volume (≈5 L) reservoir, it is also suitable for simultaneous immersion of multiple LIGPs (≈10 pieces) for only 30 min. The follow‐up hot pressing process provides a single‐step manner to stack, bond, and cure multiple preimpregnated LIGP layers in certain molds. Two processing paths then determine the 3D macrostructure with varied shapes. The first one relies on the use of two plates to construct flat‐shaped LIGP‐C before launching a high‐powered (5 W) laser cutting process. The second one directly applies 3D‐printed molds to construct curve‐shaped LIGP‐C. Detailed assembly method is displayed in Figure S1b (Supporting Information). To evidence the above points, a 20 × 20 × 0.2 cm^3^ sized LIGP‐C was first demonstrated with 20 LIGPs stacked together in flat molds (Figure 1b and Figure S1c, Supporting Information). For comparison, a neat LIGP without resin impregnation and a single‐layered LIGP‐C with the same areal dimension are shown, respectively, in Figure 1c and Figure S1c (Supporting Information). Different from the neat LIGP with curly, rough, and flexible features, the single‐layered LIGP‐C obviously becomes smoother and stiffer. With high‐thickness structure, furthermore, the 20‐layered LIGP‐C is even more rigid in which it could hold its flatness when standing on a fingertip. These observations all indicate the uniform process of resin infiltration for enabling strong connections among graphene particles. Along with mechanical reinforcement, the processed surface area (not limited to 400 cm^2^) has far exceeded the ones using template support or on‐site lay‐up with only ≈1 cm^2^.^[^ ^47^, ^48^ ^]^ Meanwhile, the scalable lamination thickness was also demonstrated by stacking various layers from 1 (135 µm) to 30 (3400 µm) (Figure 1d), proving a wide geometrical range. Benefiting from hybrid laser cutting with programmable scribing path, Figure 1e demonstrates three flat‐shaped LIGP‐C including disk, pentagram prism, and Chinese knot structures. Assisted by varied 3D‐printed molds, similarly, Figure 1f accordingly shows three curve‐shaped LIGP‐C with arch‐bridged, wavy, and ring‐like structures. Compared with previous methods, the developed process is truly effective for improving the construction of 3D graphene macrostructures with improved processing efficiency, size scalability, and shape customizability, which is highly potential for satisfying the fast/large manufacturing of future devices with wide selection of dimensional requirements.

To advice the final formation of LIGP‐C with the best quality, structural characterization of the raw materials, i.e., the neat LIGP is extremely important to understand its structural evolution under varied processing conditions. Previous research has demonstrated that the LIGP derived from a commercial polyimide paper (PI paper) is the result of photothermal transformation based on a CO_2_ laser processing with specific wavelength of ≈10.6 µm.^[^ ^44^ ^]^ Namely, localized laser irradiation with high temperature causes the breaking of C—O, C═O, and N—C bonds on PI paper and the sublimed atoms are recombined to form porous graphitic structures with rapid release of gaseous products. During this reaction, the energy level of laser irradiation determines the microstructure of graphene. Generally, the bigger the pores and the finer the skeleton structure, the higher the quality of graphene is formed.^[^ ^49^ ^]^ In the supplementary scanning electron microscope (SEM) images, Figure S3 in the Supporting Information indeed shows that higher laser power (from 1 to 1.15 W) tends to increase the level of porosity while relatively low power (0.95 W) is not enough to initiate the carbonization process for producing the porous network. Raman spectra in Figure S4 (Supporting Information) further compare the graphitization degree of LIGPs obtained from 1 to 1.15 W. Specifically, the D peak at 1350 cm^−1^ is related to existence of vacancies and bent sp^2^ bonds; the G peak at 1580 cm^−1^ is originated from a first‐order inelastic scattering process and the 2D peak at 2700 cm^−1^ is induced by second‐order zone‐boundary phonons.^[^ ^38^ ^]^ As the power gradually enhances, the intensity ratio of D peak to G peak (*I*_D_/*I*_G_) decreases significantly from 1.03 to 0.62 while that of 2D peak to G peak (*I*_2D_/*I*_G_) almost maintains at ≈0.54 (Figure S4, Supporting Information), indicating the best‐quality graphene processed under 1.15 W laser.^[^ ^4^, ^38^ ^]^ When the power reaches 1.2 W, nevertheless, multiple visible defects as well as an obvious crack have been found (Figure S3f, Supporting Information), suggesting strong ablation effect on the LIGP when processing power is too high. In brief, the power from 1 to 1.15 W can be considered as the optimum range of laser irradiation for processing the 3D LIGP‐C.

**Figure 2:**
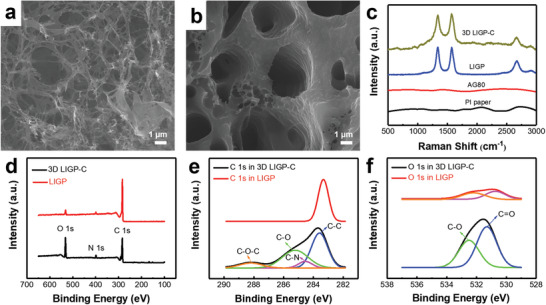
SEM images of a) LIGP and b) 3D LIGP‐C. c) Comparison of Raman spectra of pristine PI paper, AG80 epoxy resin, LIGP, and 3D LIGP‐C. d) XPS surveys, e) C 1s XPS spectrum, f) O 1s XPS spectrum of LIGP and 3D LIGP‐C.

Compared with neat LIGPs, both microscopic network and elemental content could be altered upon resin impregnation and hot pressing with infiltrated and crosslinked resin molecules. To study the differences, SEM, Raman, and X‐ray photoelectron spectroscopy (XPS) were explored. As a representative 1 W processed LIGP‐C specimen with 10‐layered structure and 15 wt% resin, SEM images show clearly that the fine‐skeleton‐like porous network in the neat LIGP (**Figure** **2**a) has been partially filled after resin infiltration and curing, indicating the formation of LIG/epoxy composite structures in the 3D LIGP‐C (Figure 2b). To verify if the resin hybridization could influence the original quality of LIG, Raman shifts were compared. As shown in Figure 2c, in contrast to PI paper and cured AG80 with featureless Raman signal, both LIGP and LIGP‐C show the three similar characteristic peaks for representing intrinsic features of graphene. These well confirm that the interfusion of resin molecule only introduces physical crosslink between LIG and AG80 without altering the original bonding structure of the LIG network. In addition to Raman, XPS (Figure 2d) further analyzed the element content of LIGP and LIGP‐C, showing obvious degradation in the C/O ratio after resin infiltration. Specifically, with the carbon content reduced clearly from 93.1% to 74.9% (Figure 2e) and the oxygen content enhanced notably from 4.6% to 24.7% (Figure 2f), the formation of interfused LIG/epoxy structure has been further confirmed after the processes of resin impregnation and hot‐pressing.

### 2.2 Process‐Determined Multifunctional Characteristics of Single‐Layered LIGP‐C

**Figure 3:**
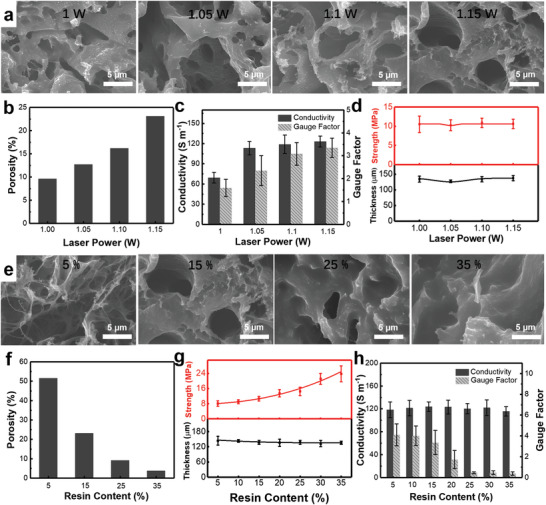
Tuning performance of single‐layered LIGP‐C. a) SEM images of single‐layered LIGP‐C irradiated under varied laser powers: 1, 1.05, 1.1, 1.15 W. Comparison of b) porosity, c) conductivity and gauge factor, d) tensile strength (top) and thickness (bottom) of single‐layered LIGP‐C processed under different laser powers. e) SEM images of single‐layered LIGP‐C obtained from different resin contents: 5, 15, 25, 35 wt%. Comparison of f) porosity, g) tensile strength (top) and thickness (bottom), h) conductivity and gauge factor of single‐layered LIGP‐C obtained from different resin contents.

Benefiting from the combined laser irradiation and resin infiltration protocol, the processed 3D macrostructures with continuous graphene and epoxy hybrid network is highly expected to perform multifunctional physical characteristics, including mechanical, electrical, and piezoresistive properties. As the fundamental structural element, accordingly, the single‐layered LIGP‐C was systematically studied first for tuning and optimizing its multifunctionality through controlling various adjustable processing parameters, including resin type, laser power, and resin content. Considering the critical role of resin for effectively strengthening the LIG network, selection of a suitable resin system could be a prerequisite for supporting the 3D LIGP‐C with the best tuning effect. Thus, as three representative commercially available epoxy resins, IN_2_, E51, and AG80 were applied, respectively, to prepare single‐layered LIGP‐C processed with the same irradiation power (1 W) and resin content (15 wt%). By applying four‐point probe, static tensile and coupled tensile‐resistance monitoring tests, electrical conductivity (EC), tensile strength (*σ*_TS_), and gauge factor (GF) of varied LIGP‐C have been quantified. When compared with the neat LIGP, although all the three cured laminates reflect similar reduction of both EC and GF from 122 ± 0.2 S m^−1^ and 4.15 ± 0.36 to ≈70 S m^−1^ and ≈1.6 caused by the infiltrated resin for introducing slight destruction of the conducting network, mechanical reinforcement of the LIGP/AG80 laminate as shown in Figure S5 (Supporting Information) reflects the strongest effect for improving *σ*_TS_ from 4.2 to 12.2 MPa where the *σ*_TS_ of LIGP/IN_2_ and LIGP/E51 are only 7.3 and 10.1 MPa. By confirming the properties of every pure resin cured structures (Figure S6, Supporting Information), the best mechanical improvement of the LIGP/AG80 laminate could be attributed to the high crosslink density of AG80 with low viscosity (≈3.2 Pa s at 30 °C).^[^ ^50^ ^]^ Thus, AG80 epoxy is ideal for constructing 3D LIGP‐C. By fixing the resin recipe, laser power as the key parameter for determining the irradiated energy density was then investigated for correlating the properties of single‐layered LIGP‐C with the same resin content (15 wt%). As the increase of laser power from 1 to 1.15 W, **Figure** **3**a first displays the microscopic SEM images of all samples, revealing the interfused LIG/resin structure with monotonically increased porosity from 9.6% to 23.1% (Figure 3b). Along with the evolutive microstructure, both EC and GF are accordingly enhanced from 69 ± 8.0 S m^−1^ and 1.59 ± 0.37 to 123 ±8.1 S m^−1^ and 3.35 ± 0.42, as the increase of laser power (Figure 3c). The observed ascending trends of porosity and EC have agreed with previous findings that higher irradiated power introduces higher‐quality graphene conversion but promotes the ablation effect.^[^ ^42^, ^44^ ^]^ Additionally, the higher‐leveled power could also enlarge the heat affected area to form overlapped processing region, which induces repeated irradiation to further improve the graphitization level.^[^ ^46^, ^51^ ^]^ As for GF, the ablation‐induced high‐porous structure could introduce the LIG conducting network with large numbers of microfissures or microcracks.^[^ ^51^, ^52^, ^53^ ^]^ Under the same mechanical deformation, the LIGP‐C with more structural defects in the micron scale would cause greater disruption of tunneling resistance when stress concentration distributes on more local points, thereby leading to higher GF. In addition to the positive effect on EC and GF, Figure 3d further evidences that both *σ*_TS_ and thickness of LIGP‐C almost keep identical at ≈10.6 MPa and ≈135 µm under various processing level, suggesting that the incorporated resin instead of laser power is the key factor for inducing the reinforced mechanical performance of LIGP‐C. Considering the optimal electrical and piezoresistive performance along with high strength, the 1.15 W laser was then selected as the ideal condition for subsequent LIGP‐C manufacturing.

Previous modulations of raw materials and processing condition strongly hint that the resin content could play a critical role on mechanical reinforcement. Thus how this factor influences the multifunctionality of LIGP‐C was further studied. As confirmed in Figure 3e, the microporous network of LIG has been gradually filled as the increase of AG80 from 5 to 35 wt% with 1.15 W laser processing, indicating that more resin content has actually been incorporated in the composite structure with reduced porosity from 51.6% to 3.8% (Figure 3f). By maintaining the thickness at ≈135 µm, Figure 3g indeed represents the prominent and continuous reinforcement of mechanical strength *σ*_TS_ from 8.1 ± 1.4 to 23.7 ± 4.2 MPa as the resin content increases. Meanwhile, Figure 3h shows that the electrical conductivity EC keeps at similar level around ≈120 S m^−1^ regardless of the resin content ranged from 5% to 35%, indicating that more resin usage would not further disrupt the conducting network of LIG. This observation is thus meaningful that high resin content could not only best reinforce but completely benefit the optimal electrical performance through high laser power processing. For piezoresistive performance GF, nevertheless, Figure 3h shows the gradual decay from 6.81 ± 2.20 to 0.39 ± 0.15 as resin increases. This can be explained that the rich resin content improves the overall integrity of the composite structure and thus weakens the concentrated local stress, thereby leading to reduced resistance change under the same deformation. In addition to the above three factors, Figure S7 (Supporting Information) further explores the effect of hot‐pressing pressure ranged from 1 to 6 MPa, presenting similar *σ*_TS_ (≈10.3 MPa), EC (≈122 S m^−1^), and GF (≈3.29) for all LIGP‐C specimens. This suggests that the hot‐pressing pressure is not a dominant factor to modulate multifunctional properties of LIGP‐C. In a brief, for single‐layered LIGP‐C, comparatively high mechanical strength (≈23.7 MPa) and electrical conductivity (≈120 S m^−1^) are simultaneously assured by relying on optimal resin type (e.g., AG80), high processing power (≈1.15 W), and rich resin content (≈35 wt%), although inevitably sacrificing its piezoresistive performance.

### 2.3 Process‐Determined Multifunctional Characteristics of Multilayered LIGP‐C with Improved Robustness

**Figure 4:**
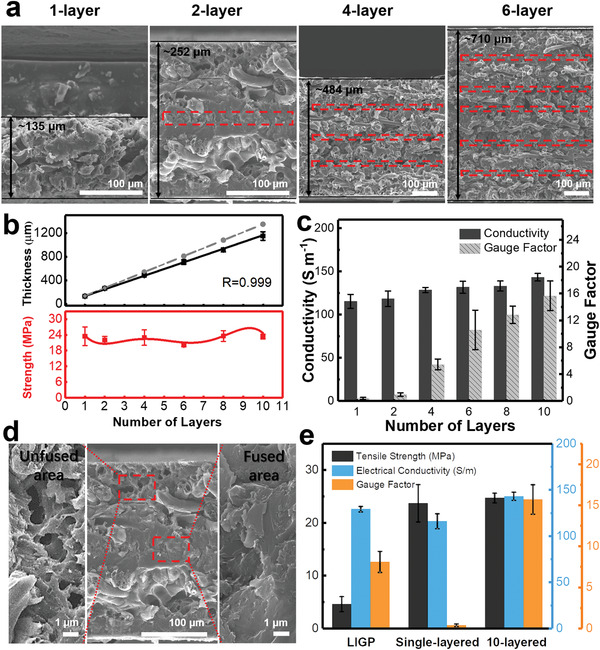
Tuning performance of multilayered LIGP‐C. a) Cross‐sectional SEM images of varied multilayered LIGP‐C from 1 layer to 6 layers where the red dashed line represents the fusion area. b) Thickness (upper panel) and tensile strength (bottom panel) of LIGP‐C with varied layers from 1 to 10. c) Comparison of conductivity and gauge factor of multilayered LIGP‐C. d) Cross‐sectional SEM images of unfused area and fused area of two‐layered LIGP‐C. e) Comparison among LIGP, single‐layered, and ten‐layered LIGP‐C with different tensile strength, electrical conductivity and GF.

Based on the solid foundation to establish and understand the process‐dependent performance of single‐layered LIGP‐C, it is then straightforward to explore the variation of this relationship while the macrostructure is thickened from single to multiple layers by stacking and hot‐pressing various sets of LIGP/AG80 prepregs from 2 to 10 pieces using optimized resin type (AG80 epoxy), laser power (1.15 W), and epoxy content (35 wt%). To evidence the possibility of property change, the cross‐sectional SEM images of varied multilayered LIGP‐C were preinvestigated. **Figure** **4**a is clearly guided that compared with the single‐layered specimen showing uniform forest‐like structure throughout its thickness direction, the double‐, quadruple‐, and six‐layered LIGP‐C, respectively, exhibit one, three, and five fusion zones (≈20 µm in thickness), in which every two neighboring sheets merge partially inside each other to form a densified morphology under the hot‐pressing process. To further verify this, Figure 4b (upper panel) reveals the thickness of LIGP‐C as a function of layered number. It is clear that as a comparison with the idealized thickness (dashed line) by direct accumulating the number of single layers (*Y =* 135 *X*, *X* is number of layers; *Y* is thickness), the experimentally determined thickness follows another linear fitting, giving *Y’* = 20 + 115 *X* with the linear correlation *R* = 0.999. The gap between the two trends (*Y–Y’* = 20 *X – 20*) then quantitatively indicates the amount of 20 µm merging depth from 0 to 9 as the stacking layer increases from 1 to 10, which agrees well with the findings in cross‐sectional SEM images.

Following the proof of structural change, it is thus critical to explore the corresponding influence of electrical, mechanical, and piezoresistive performance. Figure 4b (bottom panel) reveals the maintenance of tensile strength *σ*_TS_ at similar levels (≈24.7 MPa) when the number of layer changes from low to high. This independent behavior once again indicates that the reinforced strength is majorly contributed to the resin content instead of LIG. In a contrary, the altered LIG structure could trigger the change of electrical performance by densifying its conducting network to reduce the junction distance between LIG particles/domains.^[^ ^54^, ^55^, ^56^ ^]^ As a result, Figure 4c indeed shows an ascending trend of electrical conductivity EC from 116.8 ± 8.1 to 143.3 ± 4.4 S m^−1^ as the accumulation of LIGP‐C layer is from 1 to 10. In addition to the slightly improved EC, it is rather interesting that gauge sensitivity has significantly increased as the stacking of lamination layers (Figure 4c). Remarkably, by deforming the specimen up to 0.5% strain, the continuously improved GFs show almost an ≈40 times increase from 0.39 ± 0.15 to 15.65 ± 2.21 as the thickening of LIGP‐C from 1 to 10 layers. For example, comparing to pristine LIGP with the GF of 8.09 ± 1.28, the six‐layered LIGP‐C has reached comparable GF (10.57 ± 2.05); when the number of layer equals to 10, the GF is almost twice of the pristine one. Instead of using 35 wt% resin, similar trends of both EC and GF have been determined even if the resin content is decreased to 15 wt%, as shown in Figure S8 (Supporting Information). These findings strongly prove that the LIGP‐C with 3D‐laminated structure is prone to demonstrating ultra‐sensitive piezoresistive performance. To understand this behavior, magnified cross‐sectional SEM images as shown in Figure 4d have provided more detailed structural information. In the unfused area, the porous structure of the original LIG network is still visible even though resin particles have infiltrated inside. In contrast, the cross‐sectional surface of the fusion area clearly becomes smoother and it is difficult to trace pores or voids. Based on microscopic observations, we have accordingly deduced that two mechanisms could together determine the improved gauge sensitivity. For one thing, under hot‐pressing pressure, certain amount of resin could be squeezed out of the fusion area as the crushing and closing process of pores/voids. With less amount of resin in certain regions, the overall integrity of the structure could be influenced and it is thus easier to be disrupted by mechanical deformations. For another thing, during the fusion process, the original distribution of conducting pathways could be partially destroyed because two sets of LIG networks are merging with each other. As a result, more contact points could be generated to improve the tunneling effect of the overall conducting network with enhanced piezoresistivity.^[^ ^56^, ^57^ ^]^

With a clear comparison of *σ*_TS_, EC and GF, pristine LIGP, single‐layered, and 10‐layered LIGP‐C are demonstrated in Figure 4e as three representative structures. Once again, compared with pristine LIGP with high EC (≈129 S m^−1^) and moderate GF (≈8.1), single‐layered LIGP‐C shows much improved *σ*_TS_ (≈23.7 MPa) but with substantially reduced GF (≈0.39). Notably, only the 10‐layered LIGP‐C is able to simultaneously achieve high *σ*_TS_ (≈24.7 MPa), EC (≈143 S m^−1^), and GF (≈15.7), which is uniquely advantageous to meet the requirement of multiscenario applications.

**Figure 5:**
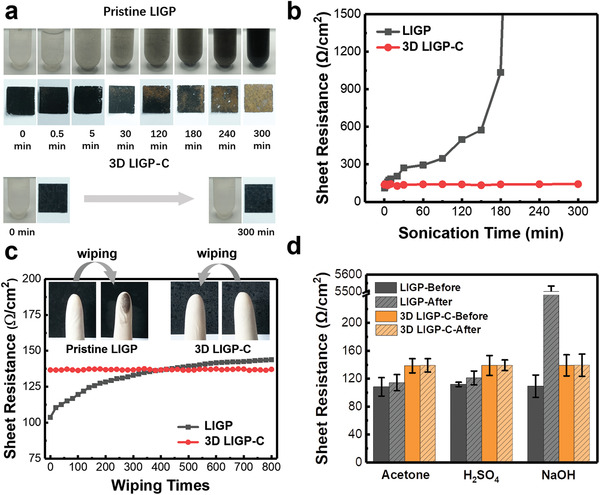
Durability tests of 3D LIGP‐C. a) Comparative demonstrations and b) resistance change between LIGP and 3D LIGP‐C treated under varied ultrasonication time. Sheet resistance changes of LIGP and 3D LIGP‐C under varied c) finger friction and d) chemical reagent immersion treatments.

In addition to the variously tunable physical properties, durability is the basis for assuring long‐time use of the proposed 3D macrostructure under varied environmental conditions. To demonstrate this point, three types of durability tests have been applied, including ultrasonic treatment, finger friction, and chemical reagent immersion. In the ultrasonic test, **Figure** **5**a compares the surface quality of pristine LIGP and LIGP‐C when subjected to a 240 W ultrasonic treatment in water for up to 300 min. For the pristine one, a series of photos clearly demonstrates the gradual change of aqueous solution from clear to dark; correspondingly, the color of the black surface continuously fades as the elongation of sonication time. This implies that the internal connection of the pristine LIGP is weak and the sonication power is able to separate and disperse the conducting particles. Comparatively, with the infiltrated resin structure, LIGP‐C is able to resist the attack from 300 min sonication by maintaining the original surface quality. As another solid evidence, Figure 5b shows the monitoring of sheet resistance. Once again, the resistance of pristine LIGP keeps ramping and it suddenly goes to infinity at 240 min indicating the total damage of conducting network. Comparatively, the sheet resistance of LIGP‐C only changes from 136.2 to 142.1 Ω cm^−2^ (less than 5%) after the 300 min ultrasonic treatment, proving the strengthened connection between particles. Compared with long‐time sonication resistance, abrasion resistance is another important factor for determining its reliability. Figure 5c shows clearly that by wiping the specimen surface using finger, the LIG particles are easily transferred to the glove for pristine LIGP and its resistance accordingly increases from 103.7 to 143.6 Ω cm^−2^ by repeatedly wiping the surface for 800 times. For the case of LIGP‐C, in contrast, the glove remains its originality and the sheet resistance also maintains its original level (≈137 Ω cm^−2^) after 800 times wiping. In addition to resisting varied physical disruptions, the ability to resist various chemical reagents is another important indicator for meeting actual applications. Specifically, pristine LIGP and LIGP‐C were, respectively, soaked in 1 m NaOH aqueous solution, 3 m H_2_SO_4_ aqueous solution, and acetone for overnight to test its resistance change in alkali, acid, and organic environment. As summarized in Figure 5d, the soaking of different chemical reagents has little effect on the sheet resistance of LIGP‐C, but it shows recognizable effects on the pristine one, e.g., its resistance has enlarged 500 times after soaking in NaOH solution. To sum up, the above tests have allusively proved that the 3D‐laminated LIGP‐C exhibits excellent durability against varied physical and chemical disruptions, suggesting a good prospect for use in multiple harsh environments.

### 2.4 3D LIGP‐C Sensor Array Enabled Smart Composites for Strain Mapping

**Figure 6:**
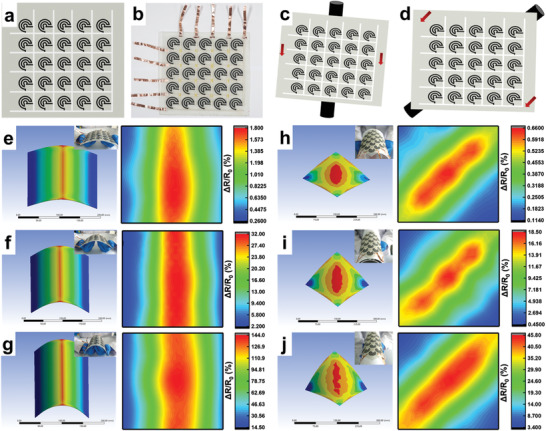
a) Schematic and b) actual images of a 5 × 5 sensing array that are uniformly distributed on a 200 × 200 × 2 mm^3^ sized FRP composites with 25 spiral‐shaped LIGP‐C elements. Schematic diagram of two typical force modes of the composites along the c) central and d) diagonal directions. Mechanical strain distribution of a deformed composites acquired by finite element analysis (left part) and resistance monitoring of 3D LIGP‐C sensor array (right part) after different maximum deflection (*ω* = 20 mm, *ω* = 40 mm, *ω* = 60 mm) by bending the square composites along its e–g) central line and h–j) diagonal direction. (The color version of this picture can be viewed online.)

Great mechanical, electrical, and piezoresistive properties as well as trimmable shape and excellent durability have endowed the possibility of 3D LIGP‐C for launching various applications. As a representative and unique case, 25 spiral‐shaped LIGP‐C elements forming a 5 × 5 sensing array was designed and mounted on top of FRP composites for mapping different strain distributions. **Figure** **6**a,b, respectively, shows schematic and actual images of the sensor array that are uniformly distributed on a 200 × 200 × 2 mm^3^ sized FRP composites with five horizontally and five vertically printed electrodes. The spiral geometry was specifically designed considering its isotropic sensing behavior when it deforms toward varied directions.^[^ ^53^, ^58^ ^]^ As shown in Figure 6c,d, two typical types of strain distribution of the laminate along the central and diagonal directions are, respectively, detected by loading different degrees of bending deformation. With the aid of finite element analysis (FEA) for revealing the symmetrically distributed strain on top of the structure surface, when FRP composites are bent with the maximum deflection of 20 mm along the central line (in inset of Figure 6e), similar distribution is found from the Δ*R*/*R*_0_ profile of LIGP‐C. The Δ*R*/*R*_0_ value gradually decreases with the increase of distance from the centerline, showing a parabolic feature in which sensor elements along the center experience the highest levels of Δ*R*/*R*_0_ up to ≈1.7% and the ones located away from the center present attenuated Δ*R*/*R*_0_ down to ≈0.39%. In addition, we also observed analogous strain and Δ*R*/*R*_0_ distributions with a parabolic feature as the maximum deflection is increased to 40 mm (Figure 6f) and 60 mm (Figure 6g). It is worth noting that the highest level of Δ*R*/*R*_0_ in the center location can reach ≈142.3% with the increased bending deformation. When we change the force loading direction from parallel to both sides to along the diagonal line, the strain distribution on the top surface determined by the FEA is also highly similar with the Δ*R*/*R*_0_ distribution. As shown in Figure 6h, when the maximum deflection comes up to 20 mm, the maximum strain and Δ*R*/*R*_0_ (≈0.63%) are distributed in or close to the diagonal area, and as the position deviates from the symmetrical diagonal line toward the lower left and upper right corners, the values gradually decrease, showing the minimum strain and Δ*R*/*R*_0_ (≈0.11%). When the maximum deflection is increased to 40 and 60 mm, the maximum Δ*R*/*R*_0_ in the center location continues to increase to ≈18.37% (Figure 6i) and ≈45.61% (Figure 6j), respectively. It can be found that the 3D LIGP‐C is able to effectively monitor the strain distribution of the deformed composite when the bending force is loaded in different directions (e.g., central line and diagonal direction) or with different maximum deflections (e.g., *ω* = 20–60 mm). Based on its ability to map local areas with scalable size and noninvasive characteristics, 3D LIGP‐C truly show considerable application prospects in the fields of smart sensing devices, structural health monitoring, and safety control of modern composite vehicles in the future.

## 3 Conclusion

In summary, we demonstrated a simple and high‐efficient method tostack and shape 3D LIG macrostructures with excellent mechanical/electrical properties and ultra‐sensitive piezoresistivity by laminating layers of LIGPs with combined resin infiltration and hot pressing. Benefiting from the processing uniqueness, the constructed 3D LIGP‐C are compatible with large area (≈400 cm^2^), high thickness (≈3.5 mm), and customizable flat/curved shapes. Besides, systematic research was explored for investigating the process‐structure‐property relationship of 3D LIGP‐C and figured out a discipline to simultaneously regulate its multifunctional properties with high‐leveled mechanical, electrical, and sensing performance. Along with excellent durability for resisting multiple harsh environments and reliable performance for strain‐mapping applications, it is expected that the established processing technology for assembling 3D LIG macrostructures is potentially suitable for satisfying the fast/large manufacturing of future devices with wide selection of dimensional requirements.

## 4 Experimental Section

### Preparation of LIG Papers (LIGPs)

Following previously established protocol,^[^ ^44^ ^]^ LIGPs were prepared by irradiating PI papers (90 µm in thickness, PolyKing Co.) as precursor materials using a 10.6 µm CO_2_ laser platform (DLS 2.3, Universal Laser Systems, Inc.). Under fixed line space (0.1 mm), pixel per inch (1000), scan rate (5.08 cm s^−1^), and certain laser power ranged from 0.95 to 1.2 W, freestanding LIGPs were formed after line‐by‐line processing both sides of the PI paper under ambient conditions.

### Preparation of LIGP/Epoxy Prepregs

Before laminating layers of LIGPs, each LIGP was impregnated with epoxy resin to prepare LIG/epoxy prepregs. AG80 resin (tetraglycidyl diaminodiphenyl methane with epoxy value 0.75–0.85) and diamino diphenyl sulfone (DDS) hardener purchased from Hubei Gemvon New Material Co. were first mixed in a weight ratio of 100:58. By using acetone as solvent, homogenous resin solutions were prepared under the treatment of sonication (JP‐040S, Shenzhen Jiemeng Cleaning Equipment Co., Ltd.) for 0.5 h with epoxy content ranged from 5 to 35 wt%. For resin impregnation, LIGPs were immersed in certain solution for 1 h and dried in an oven at 70 °C for 0.5 h. To compare the effect of different resins, E51 (bisphenol A epoxy resin with epoxy value 0.48–0.54) and IN_2_ (bisphenol A epoxy resin with epoxy value 0.23–0.38) were also used following the same protocol with curing agent BC126 (purchased from Dasen Material Technology Co., Ltd.) and AT30 hardener (purchased from Compound Material Tesco Technology Co., Ltd.).

### Preparation of 3D LIGP/Epoxy‐Laminated Composites (LIGP‐C)

The as‐prepared LIGP/epoxy prepregs were then used as raw materials for constructing 3D laminated structures. Single or multiple LIGP/epoxy prepregs were stacked together and subjected to hot press (CMP4122, Carver Inc.) with different stages of heating, including 130 °C for 1 h, 180 °C for 3 h, and 200 °C for 1 h to construct LIGP/AG80‐C. While the curing temperature was 120 °C for 3 h to construct LIGP/E51‐C and that was 60 °C for 8 h to construct LIGP/IN_2_‐C. During the hot‐pressing process, pressure was changed from 1 to 6 MPa. For shaping 3D LIG macrostructures, two processing paths were conducted to determine 3D LIGP‐C with customizable flat or curved shapes. Theone relies on the use of two plates to construct flat‐shaped LIGP‐C before launching a high‐powered laser cutting process (power = 5 W, scan rate = 1.02 cm s^−1^). The other one directly applies 3D‐printed molds to construct curve‐shaped LIGP‐C.

### Structural Characterization and Property Evaluation

SEM (JEOL JSM 7001F) was performed at 10 kV to characterize the morphologies of LIGPs and 3D LIGP‐C. Image‐Pro software was used to analyze porosity of different 3D LIGP‐C based on SEM photographs. Reflected‐light microscope (Nikon, SMZ800N) was applied to measure the thickness of different 3D LIGP‐C. A Raman spectroscope (Horiba HR800) was employed to obtain the Raman spectra of LIGPs and 3D LIGP‐C using a 532 nm laser with the power of 5 mW in which the ratio of integrated intensity of the D peak and G peak (*I*_D_/*I*_G_) and the ratio of integrated intensity of the 2D peak and G peak (*I*_2D_/*I*_G_) were calculated. XPS (Thermo Fisher ESCALAB 250Xi) was applied for comparing the content of surface elements between pristine LIGP and 3D LIGP‐C by determining the atomic percentage of C and O through the measurement of C 1s (282–290 eV) and O 1s (529–537 eV) peak intensities, respectively. To evaluate the electrical property of LIGPs and 3D LIGP‐C, resistance (*R*) of samples was measured with Keithley 2110 digital multimeter, and the corresponding electrical conductivity was quantified by Equation (1)

(1) $$\sigma \;=\;1/\rho \;=\;l/\left(R\times A\right)$$

where *A* is the section area and *l* is the electrode distance. The stress–strain curves of dog‐bone‐shaped specimen were performed with an E44.104 tensile machine (MTS Systems Corp.) for investigating the mechanical property. The tensile strength of different specimens was evaluated with Equation (2)

(2) $$\sigma_{b}=F_{b}\;/A_{0}$$

where *F*_b_ is the force that causes the fracture or a breaking and *A*_0_ is the cross‐sectional area of the testing specimens. The real‐time resistance change was synchronously monitored by coupling cyclic tensile tests with a home‐established LabVIEW program. Additionally, the gauge factor (GF) was used to describe the piezo‐sensitivity of a strain gauge by Equation (3)

(3) $$\mathrm{GF}\;=\;\left(\Delta R/R_{0}\right)/\Delta \varepsilon$$

where Δ*R*/*R*_0_ is the normalized resistance and Δ*ε* is the mechanical strain.^[^ ^53^ ^]^ FEA of an FRP substrate was also applied to simulate the strain distribution.

## Conflict of Interest

The authors declare no conflict of interest.

## Supporting information

Supporting Information

## Data Availability

Research data are not shared.
