# Supplementary material for: 3D‐Laminated Graphene with Combined Laser Irradiation and Resin Infiltration toward Designable Macrostructure and Multifunction
Source: Adv Sci (Weinh). 2022 Mar 24;9(15):2200362. doi: 10.1002/advs.202200362 (PMC9130875; doi:10.1002/advs.202200362)
Supplement: Supplementary file 1 — Supporting Information [file ADVS-9-2200362-s001.pdf]

ADVANCED SCIENCE
Open Access

Supporting Information

for *Adv. Sci.*, DOI 10.1002/advs.202200362

3D-Laminated Graphene with Combined Laser Irradiation and Resin Infiltration toward Designable Macrostructure and Multifunction

*Yan Gao, Yujiang Zhai, Guantao Wang, Fu Liu, Haibin Duan, Xilun Ding and Sida Luo**

Supporting Information

3D Laminated Graphene with Combined Laser Irradiation and Resin Infiltration toward Designable Macrostructure and Multifunction

*Yan Gao, Yujiang Zhai, Guantao Wang, Fu Liu, Haibin Duan, Xilun Ding, Sida Luo**

Y. Gao, Y. Zhai, Dr. G. Wang, F. Liu, Prof. X. Ding, Prof. S. Luo
School of Mechanical Engineering & Automation
Beihang University
No. 37 Xueyuan Road, Beijing, 100191, China
E-mail: s.luo@buaa.edu.cn

Prof. H. Duan
School of Automation Science and Electrical Engineering
Beihang University
No. 37 Xueyuan Road, Beijing, 100191, China

Keywords: laser-induced graphene, graphene papers, 3D graphene, smart composites, multifunctional sensors

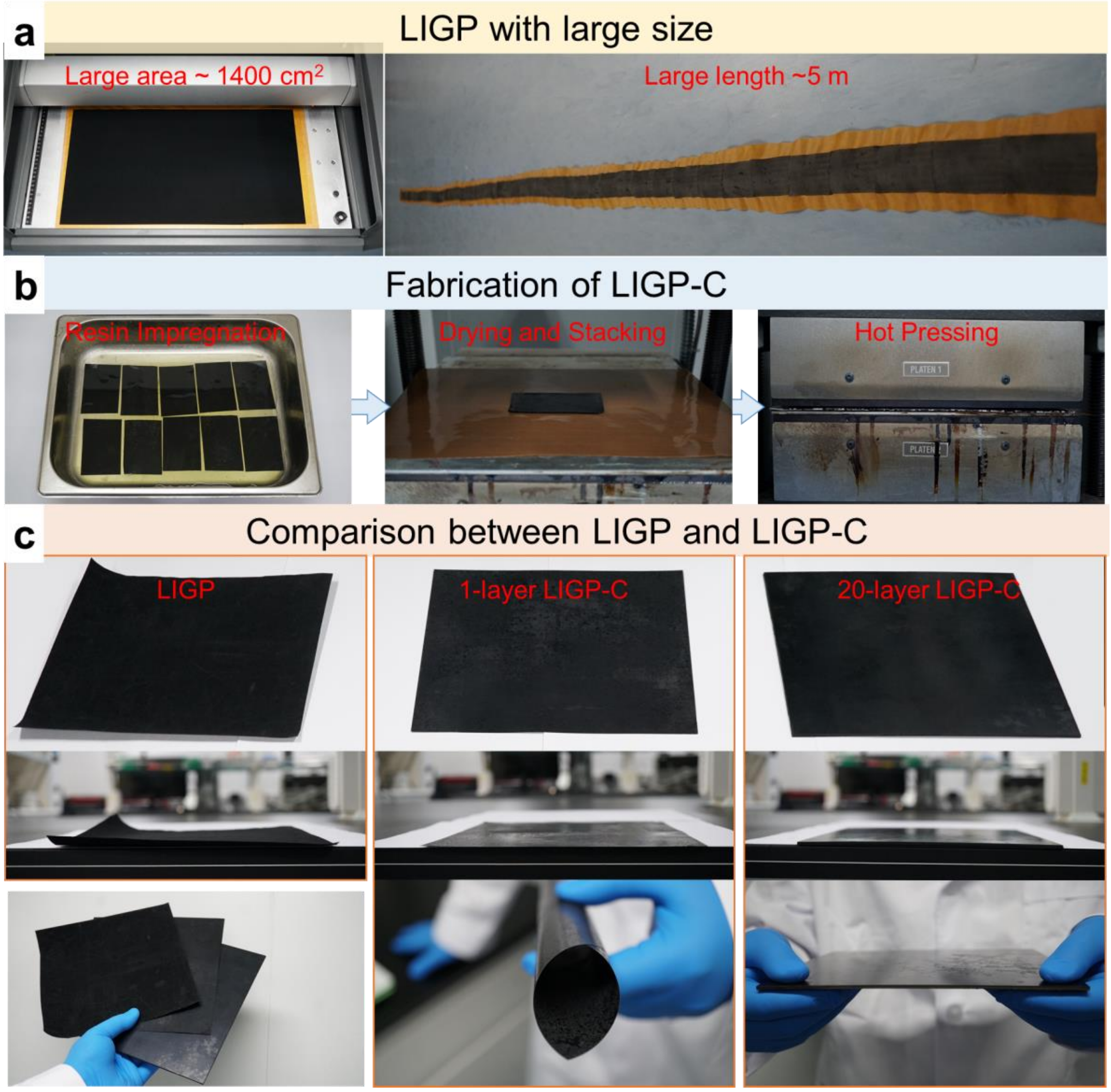

Figure S1. (a) Demonstrations of LIGP with large size (large area and large length). (b) Fabrication of LIGP-C in situ preparation including resin impregnation, drying and stacking, hot pressing. (c) Photographs of LIGP, 1-layer LIGP-C and 20-layer LIGP-C, and comparison of them in the bottom left corner.

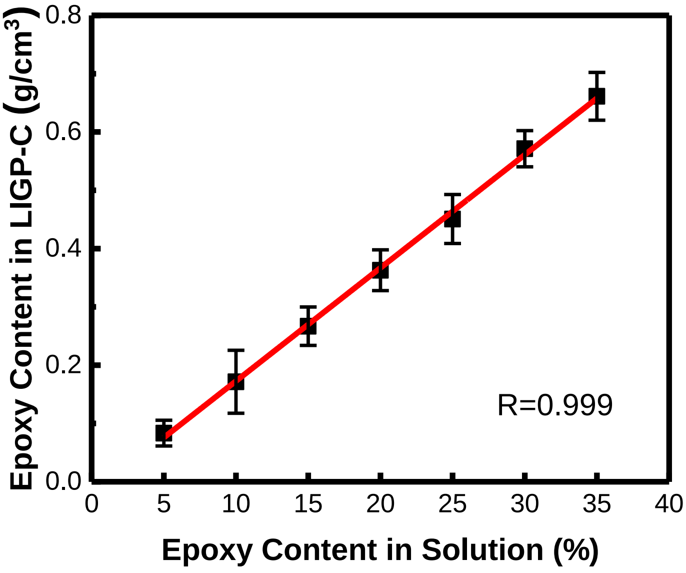

Figure S2. Actual amount of epoxy in single-layer LIGP-C obtained from different epoxy content: 5 wt% to 35 wt%. When the epoxy content in solution is 5 wt%, 10 wt%, 15 wt%, 20 wt%, 25 wt%, 30 wt%, 35 wt%, the epoxy content in LIGP-C (i.e. the mass of epoxy in unit volume LIGP-C) is 0.08±0.02 g/cm^3, 0.17±0.05 g/cm^3, 0.27±0.03 g/cm^3, 0.36±0.04 g/cm^3, 0.45±0.04 g/cm^3, 0.57±0.03 g/cm^3, 0.66±0.04 g/cm^3, respectively.

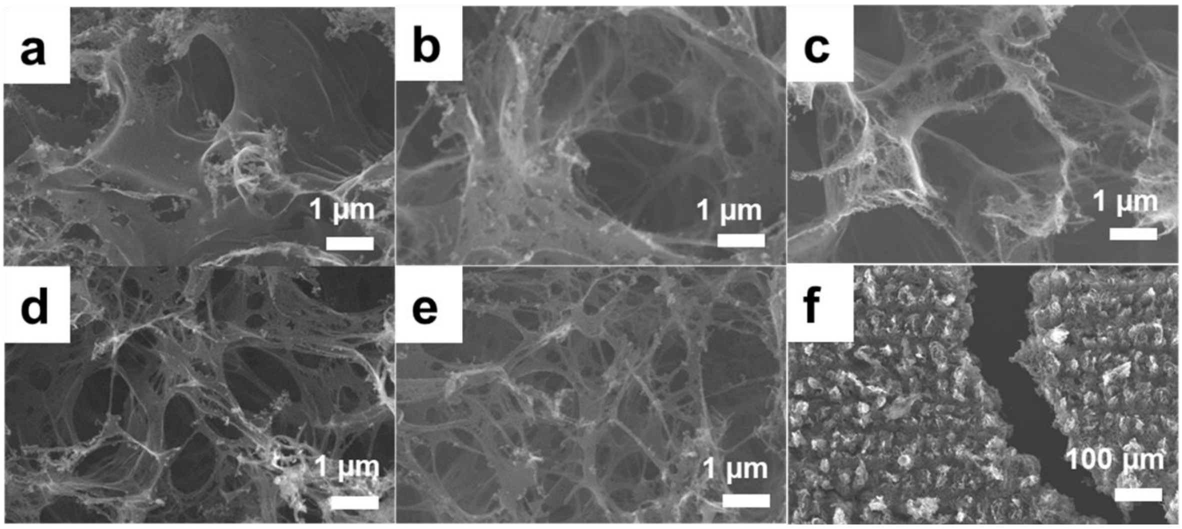

Figure S3. SEM images of LIGPs irradiated by varied powers: 0.95W (a), 1W (b), 1.05W (c), 1.1W (d), 1.15W (e), 1.2W (f).

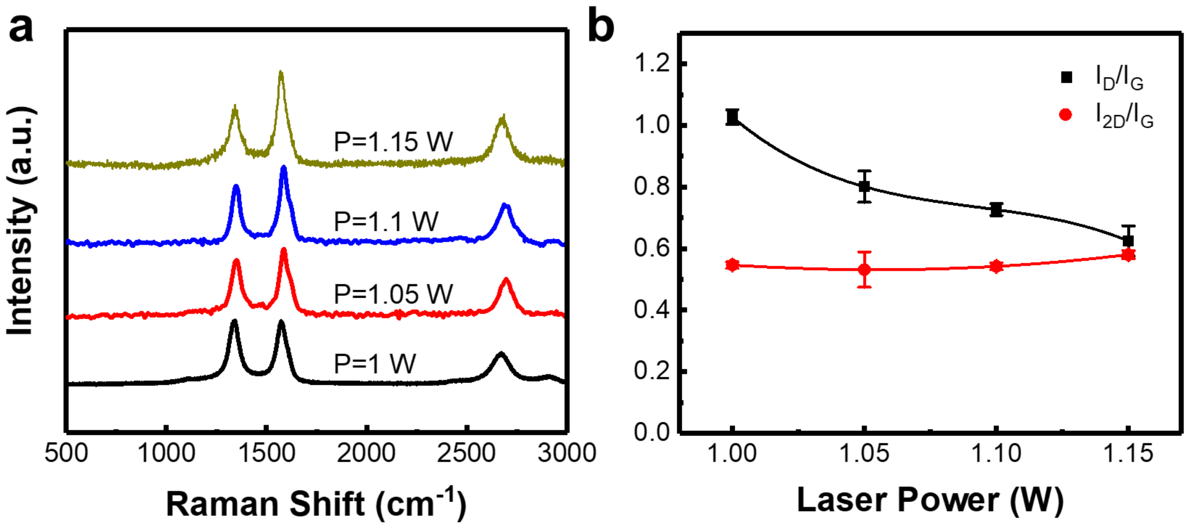

Figure S4. (a) Raman spectra of LIGPs under various laser power. (b) The statistical analysis of I_D/I_G and I_{2D}/I_G of LIGPs by varied powers laser powers.

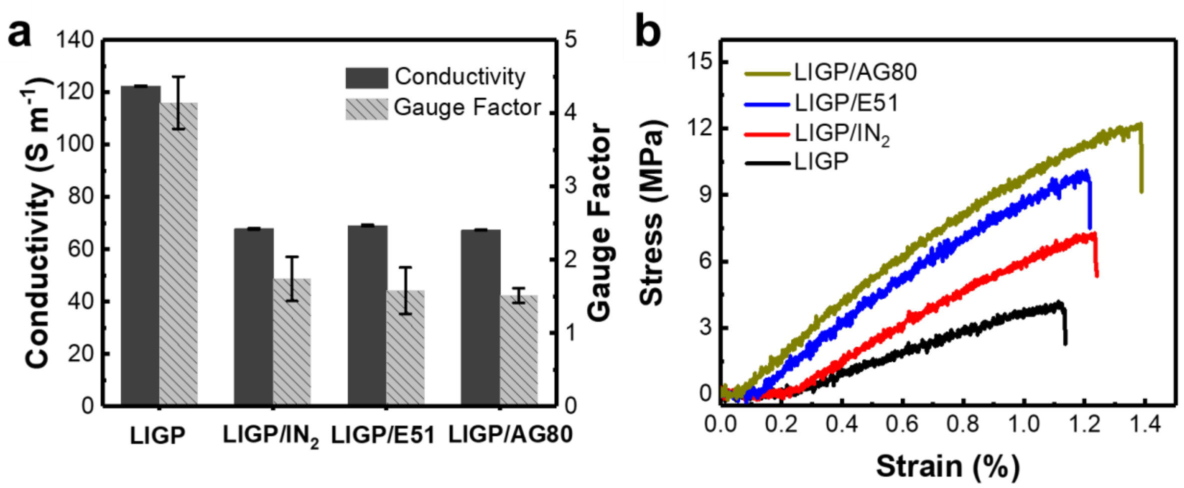

Figure S5. Comparison of conductivity and gauge factor of LIGP and single-layere LIGP/IN_2, LIGP/E51, LIGP/AG80 laminates. (b) Stress-strain curves of LIGP and single-layered LIGP/IN_2, LIGP/E51, LIGP/AG80 laminates.

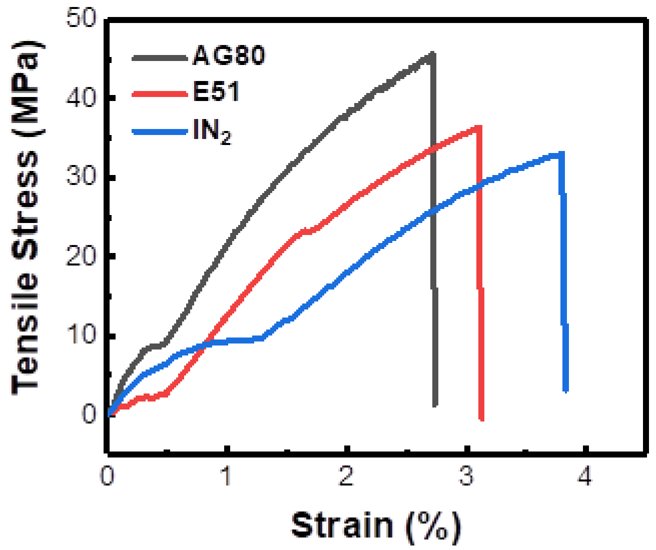

Figure S6. Comparison of tensile stress-strain curves for AG80, IN2, E51 epoxy resin.

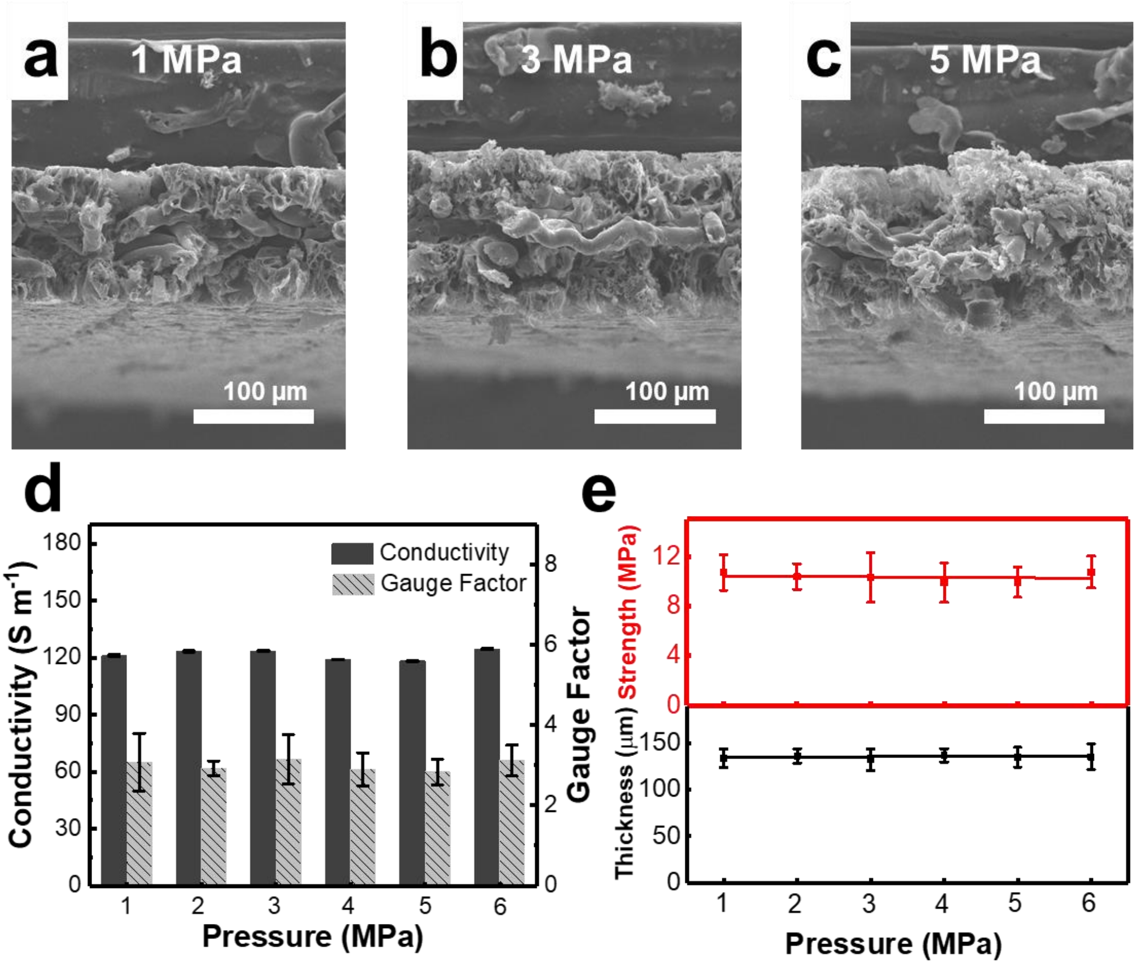

Figure S7. SEM images of single-layered LIGP-C obtained from different pressure: 1MPa (a), 3MPa (b), 5MPa (c). Comparison of conductivity and gauge factor (d), tensile strength (top) and thickness changes (bottom) (e) of single-layered LIGP-C obtained from different pressure.

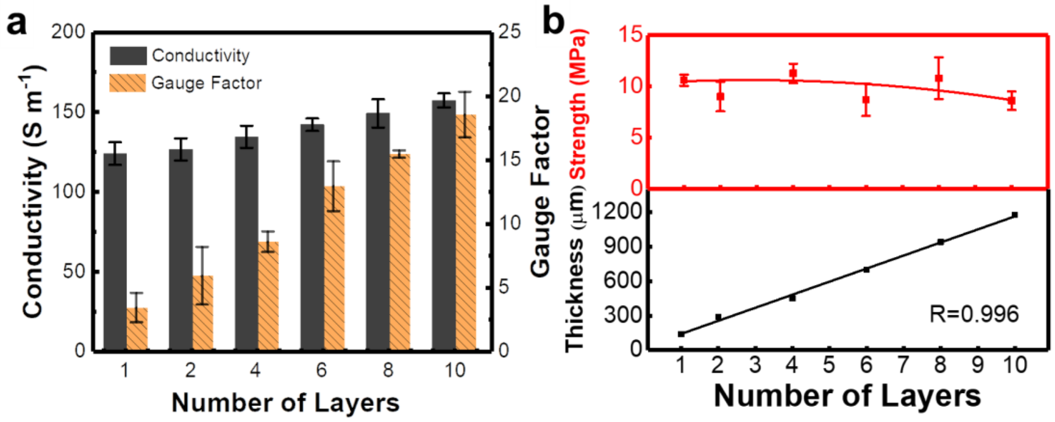

Figure S8. (a) Comparison of conductivity and gauge factor of multi-layered LIGP/AG80 laminates obtained from the resin content of 15wt%. (b) Tensile strength (top) and thickness changes (bottom) of multi-layered LIGP/AG80 laminates obtained from the resin content of 15wt%.
